# Ex Vivo Fluorescence Confocal Microscopy Meets Innovation and Revolutionary Technology, for “Real-Time” Histological Evaluation, in Pediatric Surgical Oncology

**DOI:** 10.3390/children11121417

**Published:** 2024-11-23

**Authors:** Donatella Di Fabrizio, Edoardo Bindi, Michele Ilari, Alessandra Filosa, Gaia Goteri, Giovanni Cobellis

**Affiliations:** 1 Pediatric Surgery Unit, Salesi Children’s Hospital, Polytechnic University of Marche, Via Filippo Corridoni, 16, 60123 Ancona, Italy; edoardo.bindi@ospedaliriuniti.marche.it (E.B.); michele.ilari@ospedaliriuniti.marche.it (M.I.); giovanni.cobellis@ospedaliriuniti.marche.it (G.C.); 2 Institute of Pathological Anatomy, Polytechnic University of Marche, 60123 Ancona, Italy; alessandra.filosa@ospedaliriuniti.marche.it (A.F.); gaia.goteri@ospedaliriuniti.marche.it (G.G.)

**Keywords:** ex vivo fluorescence confocal microscopy, pediatric surgical oncology, pathological imaging technology, innovation, children

## Abstract

Background and Aim: Ex vivo fluorescence confocal microscopy (FCM) systems are innovative optical imaging tools that create virtual high-resolution histological images without any standard tissue processing, either freezing or fixing in formalin and embedding in paraffin. These systems have opened an era that would revolutionize pathological examination by providing rapid, real-time assessments across various pathology subspecialties, potentially replacing conventional methods that are tissue- and time-consuming. This study aimed to present the first utilization of FCM in pediatric surgical oncology, focusing on assessing the benefits, particularly in facilitating rapid and accurate diagnosis. Methods: This preliminary study comprised five consecutive patients undergoing surgical biopsy for disease characterization and surgical strategy selection. After biopsy, tissue samples were prepared and analyzed using FCM without sectioning. A pathologist who evaluated macroscopic and microscopic images, once obtained remotely, could promptly indicate any interventions that require timeliness. Samples were then evaluated with conventional methods. Results: All five lesions were deemed suitable for evaluation. Preliminary diagnoses utilizing FCM included atypical Spitz nevus (1), Wilm’s tumor (1), lymph node reactive hyperplasia (1), malignant germ cell tumor of the testis (1), and Hodgkin’s lymphoma (1). Final histopathological analyses revealed atypical Spitz nevus (1), Wilm’s tumor (1), hyperplastic lymphadenopathy with a prevalent marginal pattern (1), mixed nonseminomatous malignant germinal neoplasm consisting of embryonal carcinoma (90%) and yolk sac tumor (10%), and Hodgkin’s lymphoma nodular sclerosis variant (1). In the case of diagnosis of atypical Spitz nevus, the widening of the resection margins was performed in the same surgery. In the case of testicular neoplasm, radical orchiectomy was performed. A high level of agreement between FCM evaluation and definitive histological examination was observed for all parameters evaluated. Conclusions: FCM represents a significant advancement in pathological imaging technology, offering potential benefits in enhancing traditional tissue processing methods. This preliminary report marks the first application of FCM in pediatric surgical oncology. Our findings underscore the promising role of FCM as an adjunctive tool in pediatric oncology, facilitating prompt diagnosis and treatment initiation.

## 1. Introduction

Childhood cancer remains the leading cause of nonaccidental death among children and adolescents [1]. Although advancements in treatment have significantly improved survival rates over the past decades, early histological diagnosis remains a critical factor in optimizing outcomes, influencing treatment effectiveness, mortality, and overall quality of life for young patients [2].

The classic histologic method of formalin-fixed and paraffin-embedded (FFPE) tissue processing, combined with hematoxylin–eosin (H&E) staining, is the gold standard for diagnosing pediatric tumors. This technique, widely established globally, offers significant advantages, such as the opportunity for immune-histochemical staining with various antibodies, molecular analyses, and the possibility of long-term tissue archiving. However, it requires approximately at least 48–72 h from specimen collection to the final diagnosis [3]. In contrast, intraoperative diagnosis on frozen sections provides a rapid histologic tissue assessment and is widely used for intraoperative consultation, particularly in oncologic surgery. However, the frozen sections technique has an overall quality far inferior to sections from paraffin-embedded tissue; the tissue used is lost for further investigation, and it is a costly procedure requiring specialized laboratory equipment, such as a cryostat, staining solutions, and appropriate workbenches, as well as a skilled technician [4].

Fluorescence confocal microscopy (FCM) is a novel digital real-time imaging technique that uses fluorophores to label specific structures within biological samples, exciting them to emit light at a longer wavelength (fluorescence reflectance). The emitted light passes through a pinhole aperture that blocks out-of-focus light, ensuring that only light from the focal plane reaches the detector (confocal reflectance). By scanning the laser across the sample and capturing images from various focal planes, FCM creates real-time, high-resolution, three-dimensional virtual H&E-like digital stain images of the sample, providing detailed visualization of cellular structures and tissues [5,6,7].

This prospective study documented our hands-on experience with the application of FCM in real-time digital imaging for rapid microscopic diagnosis of pediatric tumor specimens. The study explored the practicality and effectiveness of FCM in a clinical setting, evaluating its utility as an innovative tool for the immediate evaluation of pediatric cancers.

## 2. Methods

### 2.1. Patients

All children admitted to the Salesi Children’s Hospital requiring a surgical biopsy in order to determine the neoplastic lesion between November 2023 and March 2024 were included in the study. Demographic details, surgery, and anatomopathological information were collected prospectively during the study.

### 2.2. Confocal Microscope

In November 2023, we acquired, through a charity donation, the FCM VivaScope 2500 M-G4, developed by Mavig GmbH in Munich, Germany, and Caliber I.D. based in Rochester, NY, USA. This advanced confocal microscope is equipped with two distinct lasers that emit different wavelengths, enhancing its capability to provide detailed images of both stained and unstained tissues. The first laser operates at a wavelength of 488 nm, optimized for fluorescence imaging, which is particularly effective for visualizing acridine orange-stained nuclei. The second laser emits at 785 nm and is used primarily for reflectance imaging, allowing for clear visualization of the tissue structure.

The FCM VivaScope 2500 M-G4 offers significant depth and resolution capabilities, with a maximum examination depth of 200 microns and a vertical resolution of at least 4 microns. Additionally, the maximum scanning area of this device is 25 × 25 mm^2^, making it suitable for comprehensive scanning of larger tissue sections.

The final imaging process is achieved by stitching together multiple mosaic images, each square-shaped and consisting of 1024 × 1024 pixels. These images are captured in both grayscale fluorescence and reflectance formats, and they are digitally stained in a traditional H&E staining. This assembly is facilitated by a sophisticated built-in algorithm that accurately aligns and integrates the images.

### 2.3. Specimen and Staining Procedure

The native tissue sample was collected throughout surgical biopsy. Immediately after the sample acquisition and no later than 5 min post-harvesting, the sample was prepared. This quick processing time is crucial for preserving the cellular and molecular structures of the tissue, which is essential for accurate imaging and analysis.

The sample was first cut to sizes comparable to those typically used for frozen sections, ensuring tissue consistency, then washed with 0.9% saline solution for 10 s, focusing on the removal of blood or tissue marking.

Following preparation, the staining process was meticulously performed according to the manufacturer’s protocol to highlight cell nuclei, which are critical for detailed tissue examination.

Each sample underwent immersion in 70% liquid ethanol for 10 s, which helped to clean the tissue and prepare it for effective staining. The tissue was then stained with a solution of 0.04% acridine orange dye for 30 s, which is a potent nucleic acid selective fluorescent cationic dye useful for cellular DNA and RNA detection, providing the necessary contrast for confocal microscopy. Subsequently, the tissue was immersed in a solution of 0.067% fast green dye for 20 s, enhancing the nucleus-to-cytoplasm contrast and aiding in the recognition of the tissue’s components. Lastly, the sample was dipped in 0.9% saline solution for 10 s to remove excess dye, which could interfere with image clarity.

After staining, the tissue sample was carefully placed on a sponge and covered by magnetic-mounted glass slides to secure the setup. The sponge applies gentle pressure to ensure complete contact between the tissue and the glass, a critical step for high-quality imaging.

Before the examination process, some ultrasound gel was first applied to the lens of the device, helping to create a clear contact medium between the FCM lens and the tissue, facilitating optimal light transmission and image clarity. The prepared slide was then positioned on the FCM stage, and the laser was activated for image acquisition.

To begin the imaging process, specific settings such as the required depth, area, and the desired mode of interest had to be manually selected by the operator. Once configured, the FCM commenced scanning the tissue sample. For a standard sample size of 8 × 8 mm^2^, the scanning process took approximately 50 s. It is important to note that the scanning time is directly proportional to the size of the tissue sample; larger samples require longer scanning periods due to the increased area that the laser needs to cover.

The images acquired through this process are capable of being magnified up to 550×, the same level of magnification typically achieved in traditional histology, allowing for detailed examination of cellular structures and abnormalities. Furthermore, FCM offers the advantage of vertical scanning capabilities, allowing the effective visualization of all the layers of the tissue, from the superficial down to the deeper layers, providing a three-dimensional comprehensive view essential for thorough diagnostic assessments.

Following this imaging phase, the samples were immediately placed in biocassettes and fixed in buffered formalin. This fixation preserved the tissue for the subsequent conventional histopathological examination and evaluation.

### 2.4. Image Evaluation

All images captured during the analysis were named and stored. The images were subjected to a meticulous review process conducted remotely by two experienced pathologists to ensure comprehensive and unbiased analysis. The pathologists checked each digital image for any signs of structural defects and for the color distribution to ensure clarity and detail comparable to traditional methods. They assessed a morphological analysis scrutinizing the architecture of the tissue, nuclear morphology, and overall cellular structure to ensure that the digital images provided a clear representation of the histological features necessary for accurate diagnosis. Finally, a conclusive diagnosis of the tumor was carried out in order to start the therapeutic treatment quickly.

### 2.5. Definitive Histologic Evaluation

Tissue samples were processed using FFPE method, then sectioned into thin slices and stained using the hematoxylin and eosin (H&E) technique and examined under a microscope following the standard diagnostic morphology criteria for different pathologies.

## 3. Results

During the study period, five patients were included in the analysis, with a gender distribution of three males (60%) and two females (40%). The mean age of the patients was 10.8 years, ranging from 5 to 15 years. The surgical interventions were the following. One patient underwent excision of an atypical nevus located in the right axillary region. Another patient had an ultrasound-guided needle biopsy of a right kidney tumor, suspected to be a Wilms’ tumor and unresponsive to chemotherapy. A third patient had a right latero-cervical lymphadenopathy excised. A fourth patient required an orchidectomy for a left testicular mass. The fifth patient with a mediastinal mass underwent complete excision of a right supraclavicular lymphadenopathy.

**Figure 1:**
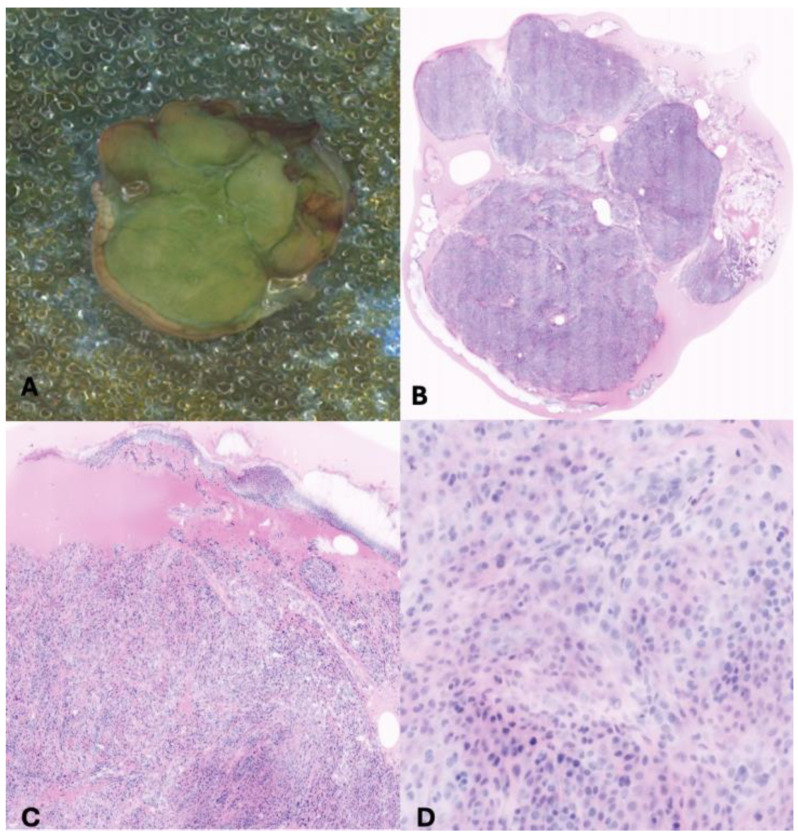
Skin lesion was excised by pediatric surgeon and the specimen was divided in three sections and colored with fluorescent dyes (**A**: one section). Vivascope scanned each section of the lesion, and the virtual slides were acquired with different grades of magnification (**B**–**D**).

All five samples were suitable for evaluation with FCM. After surgical excision, each tissue sample was processed and analyzed using FCM. Preliminary diagnoses made using FCM included atypical Spitz nevus (1, Figure 1), Wilms’ tumor (1), lymph node reactive hyperplasia (1), malignant germ cell tumor of the testis (1), and Hodgkin’s lymphoma (1). The average processing time was 220 s, with a range from 140 to 400 s. Processing time decreased notably as the study progressed, reflecting improvements in the procedural learning curve.

**Figure 2:**
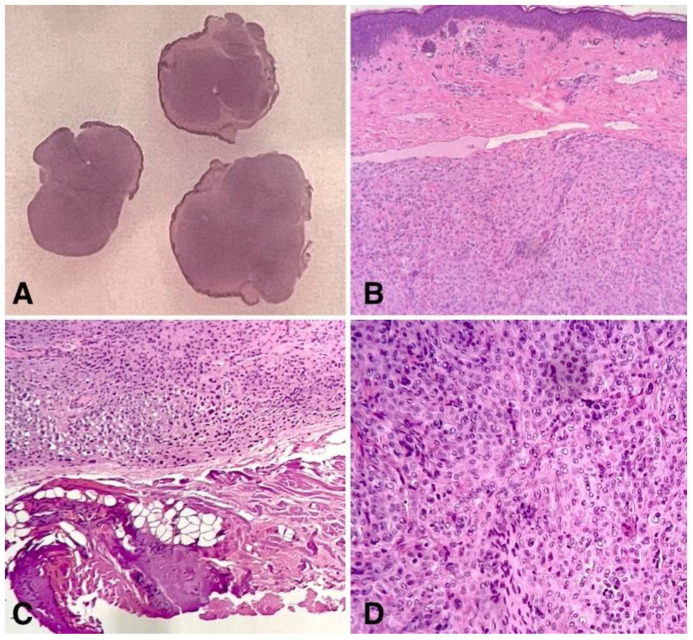
Low power slide scan: the lesion was originally cut in three sections, which were examined using confocal microscopy. Then, the same sections were put in the block for processing. The silhouette of the lesion is dermal-based (**A**). An intermediate power view demonstrates the sparing of the epidermis by the lesion, which showed a well-circumscribed superficial border and a clear-cut margin at the bottom without extending into the ipodermal fat (**B**,**C**). Cellular composition of the lesion, characterized by epithelioid plumped cells with wide eosinophilic cytoplasm, along with cytological nuclear details, were perfectly preserved at the definitive staining after confocal microscopy examination (**D**).

Final histopathological analysis confirmed the diagnoses as follows: atypical Spitz nevus (1, Figure 2), Wilms’ tumor (1), hyperplastic lymphadenopathy with a prevalent marginal pattern (1), mixed nonseminomatous malignant germinal neoplasm consisting of embryonal carcinoma (90%) and yolk sac tumor (10%), and Hodgkin’s lymphoma nodular sclerosis variant (1). In the case of atypical Spitz nevus, an extension of the resection margins was performed during the same surgical procedure based on FCM findings.

There was agreement between the FCM evaluations and final histopathology for all samples, demonstrating strong concordance across the two techniques.

## 4. Discussion

Digital pathology has emerged as a transformative technology across various medical fields, offering rapid, high-quality visualization methods that enhance both patient care and the efficiency of healthcare systems. By leveraging artificial intelligence and expanding telemedicine, digital pathology opens new avenues for improving diagnostic accuracy and patient outcomes [8]. However, to fully realize its potential, it is crucial to explore both the capabilities and limitations of these technologies.

In pediatric oncology, the speed and accuracy of diagnosis are paramount for timely and effective treatment. Traditional diagnostic methods, while considered the gold standard, often come with limitations such as extended processing times and potential compromises in tissue integrity. Our study presents an innovative, simple, ultra-fast, and safe approach for diagnosing pediatric tumors, offering improved sensitivity compared to conventional methods.

FCM represents a significant advancement in morphology-based diagnostic technology since it operates in real time, scanning fresh tissue samples directly without the need for traditional processing steps such as fixation, embedding, and sectioning. This immediate approach enables pathologists to make an informed diagnostic decision directly in the clinical or surgical setting. Furthermore, since it does not require any alteration or special preparation of the tissue, the integrity and physical state of the samples are preserved, ensuring the possibility to also analyze the same tissue sample with conventional pathology workflows, such as FFPE processing and H&E staining, without any compromise in quality. Moreover, FCM can create 3D images of samples through a process known as optical sectioning, which captures images from multiple focal planes to reconstruct a detailed three-dimensional representation of the tissue. This capability improves the visualization of spatial relationships among cells, highlighting tissue architecture that may be hidden in two-dimensional sections. This capability makes it an exceptionally valuable tool in settings where time is critical, such as in the assessment of surgical margins during oncologic surgeries or in the rapid evaluation of biopsy samples [9,10].

Recent studies underscore the growing versatility and effectiveness of FCM [11,12,13,14,15,16,17,18,19,20,21].

Initially used for skin carcinomas [11,12,13,14], FCM has expanded its applications to include a variety of tissues, such as those from the prostate, breast, thyroid, gastrointestinal tract, liver, brain, lung, and kidneys [15,16,17,18,19,20,21]. These exploratory studies and small clinical series have shown promising results, highlighting FCM’s potential for real-time, high-resolution imaging, allowing immediate diagnostic assessments and intraoperative decision-making.

A study by Villarreal et al. [19] highlighted a strong correlation between FCM images and traditional H&E stains in nephrology, affirming FCM’s diagnostic reliability. Longo et al. [13] explored the effectiveness of ex vivo FCM in the context of Mohs surgery for basal cell carcinoma (BCC), providing a substantial dataset for evaluation, revealing that, compared with traditional frozen section analysis, the diagnostic sensitivity and specificity achieved through FCM were 80% and 96%, respectively. Marenco et al. [22] and Rocco et al. [23] reported a high agreement between diagnoses derived from H&E-like digital images and conventional H&E-stained slides for prostatic biopsies, as indicated by the kappa (0.81 and 0.84, respectively).

Ragazzi et al. [7] investigated the efficacy of FCM across a diverse range of surgical fresh tissue samples from various organs, showing that the FCM images were capable of clearly distinguishing neoplastic tissues from normal tissues. The ability to differentiate between cancerous and healthy tissues in real time offers considerable benefits for precision oncologic surgery that ensures the complete removal of cancerous tissues while preserving as much healthy tissue as possible.

In our study, the effectiveness of FCM was evaluated across various surgical tissue samples from different organs. The results demonstrated FCM’s capability to accurately distinguish between neoplastic and normal tissues, thereby facilitating real-time diagnosis during the surgical procedure. We performed confocal microscopy in one case of an atypical Spitz tumor, in which the clinical appearance of the lesion was not clear. Histopathological diagnosis of melanocytic lesions is usually challenging in the pediatric population, especially when spitzoid features are predominant. Spitzoid lesions in children are frequently atypical and need a further conservative excision due to the unpredictable biological behavior of the lesion itself. Confocal microscopy could immediately suggest the spitzoid nature of the lesions, offering the pediatric surgeon the possibility of a wide excision as the first-choice approach. The pathologist can perform adequate sectioning of the skin specimen in order to examine the entire lesion with confocal microscopy and then submit the section obtained to traditional processing without compromising the integrity of the lesion and performance of conventional microscopy. In our series, we employed FCM to also evaluate core-needle biopsies, which typically provide smaller tissue samples and may be subject to trauma during the sampling process. One of the key advantages of FCM is its ability to assess in real time the adequacy of the biopsy material before proceeding with a detailed analysis. Furthermore, the high-resolution imaging enabled us to conduct a comprehensive evaluation of cellular characteristics and diagnosis of the Wilms’ tumor.

Krishnamurthy et al. [9] assessed the feasibility of ex vivo FCM examination of breast, lung, kidney, and liver samples, reporting the capability of producing good-quality images within a quick timeframe of 5 to 10 min. This rapid processing time is particularly advantageous in surgical environments, where time is often critical, and the level of detail is sufficient for the accurate categorization of the specimens for precise diagnostic assessments.

In our study, the processing time for tissue examinations varied from 400 s during the initial procedures to 140 s for the later cases. This quick processing time, improved by the learning curve, was valuable for the diagnostic speed and the effective treatment of children.

Training and proficiency in FCM are also key factors in its successful implementation. Panarello et al. [24] reported that the learning curve associated with FCM appears to be relatively short, showing that medical professionals can quickly become proficient in interpreting FCM images. Furthermore, Kose et al. [14], performing an international three-center training study in reading the specimens of BCC, found that higher sensitivities and specificities are associated with more experienced practitioners of FCM, emphasizing that proficiency improves with experience.

In order to aid pathologists in familiarizing themselves with FCM images, Bertoni et al. [25] published an atlas describing the basic features observed in prostate lesions with FCM, aiming to familiarize clinicians with the characteristic images produced by FCM and facilitate more accurate assessments.

Therefore, the rapid adaptability to FCM imaging is encouraging, suggesting that with a relatively short training period, surgeons and clinicians can effectively use this technology with the diagnostic collaboration of pathologists.

Future perspectives of FCM include telepathology and teaching processes. Since FCM digitizes the tissue samples into high-quality mosaic images, these can be sent electronically with ease, allowing pathologists to perform timely diagnostics without the need for a physical presence and facilitating quicker decision-making and more efficient patient care. The digital nature of FCM images ensures that they can be easily shared and reviewed by specialists, regardless of geographical barriers, making it an invaluable tool in modern dermatological and surgical practices [5,26].

The adoption of digital pathology also has implications for education. FCM’s modern appeal can attract students and enhance learning experiences through readily available digital images. The technology supports self-study and remote learning, making it an effective tool for teaching and training the next generation of pathologists [5].

While FCM offers several advantages in the diagnosis of pediatric tumors, it is essential to acknowledge its limitations to provide a balanced perspective on its utility in clinical practice.

One limitation of fluorescence confocal microscopy (FCM) is photobleaching, in which prolonged laser exposure causes degradation of fluorescent labels and a decrease in signal intensity over time. To mitigate this issue, establishing standardized imaging protocols can help reduce exposure time, ensuring consistency and reliability across different cases.

Another limitation relates to the resolution capabilities of FCM. While it offers a resolution of approximately 4 nanometers, this is still lower than the sub-200 nanometer resolution achieved by advanced light microscopy techniques, reflecting the trade-off for rapid real-time diagnosis.

Lastly, while FCM provides powerful visualizations of dynamic processes in real time, it does not always yield the same level of quantitative data as traditional histopathological methods, but the integration of FCM with traditional histopathological methods can provide a more comprehensive evaluation, enriching and improving the diagnostic process.

## 5. Conclusions

Digital pathology, particularly through the application of FCM, offers substantial benefits in diagnostic accuracy, efficiency, and patient care. Its ability to provide real-time, high-resolution imaging, coupled with advancements in telepathology and educational tools, positions it as a transformative technology in both clinical and academic settings. As research and technology continue to evolve, the full potential of FCM and digital pathology will increasingly contribute to improved outcomes and advancements in medical practice.

## Data Availability

Data are available on reasonable request to the corresponding author. The data are not publicly available due to ethical restrictions.
